# Structure-Function Relationship in Keratoconus: Spatial and Depth Vision

**DOI:** 10.1167/tvst.12.12.21

**Published:** 2023-12-27

**Authors:** Preetam Kumar, Peter Campbell, Pravin K. Vaddavalli, Christopher C. Hull, Shrikant R. Bharadwaj

**Affiliations:** 1 Department of Optometry and Visual Science, School of Health and Psychological Sciences, City, University of London, Northampton Square, London, United Kingdom; 2 Brien Holden Institute of Optometry and Vision Sciences, L. V. Prasad Eye Institute, Banjara Hills, Hyderabad, Telangana, India; 3 Prof. Brien Holden Eye Research Centre, Hyderabad Eye Research Foundation, L. V. Prasad Eye Institute, Banjara Hills, Hyderabad, Telangana, India; 4 The Shantilal Sanghvi Cornea Institute, L. V. Prasad Eye Institute, Banjara Hills, Hyderabad, Telangana, India

**Keywords:** contrast sensitivity, corneal topography, keratoconus, logistic regression, optical degradation, stereoacuity, structure-function relationship

## Abstract

**Purpose:**

The purpose of this study was to determine changes in spatial and depth vision with increasing severity of keratoconus and to model the structure-function relationship to identify distinct phases of loss in visual function with disease severity.

**Methods:**

Best-spectacle corrected, monocular high-contrast visual acuity, contrast sensitivity function (CSF) and stereoacuity of 155 cases (16–31 years) with mild to advanced bilateral keratoconus was determined using standard psychophysical tests. Disease severity was quantified using the multimetric D-index. The structure-function relationship was modeled using linear, positive exponential, negative exponential, and logistic nonlinear regression equations.

**Results:**

The logistic regression model explained the highest proportion of variance for spatial vision, without bias in the residual plots (*R*^2^ ≥ 66%, *P* < 0.001). Visual acuity showed a distinct ceiling phase and a steeper loss rate with increasing D-index (1.8 units/D-index) in this model. The area under the CSF lacked this ceiling phase and had a shallower loss rate (0.28 units/D-index). Stereoacuity loss with D-index was poorly explained by all models tested (*P* ≤ 0.2). Cases with lower and bilaterally symmetric D-index had better stereoacuity (181.6-376 arc seconds) than those with higher D-index (>400 arc second); both were significantly poorer than controls (approximately 30 arc second).

**Conclusions:**

Vision loss in keratoconus varies with the visual function parameter tested. Contrast sensitivity may be an earlier indicator of spatial vision loss than visual acuity. Depth perception is significantly deteriorated from very early stages of the disease.

**Translational Relevance:**

The study outcomes may be used to forecast longitudinal vision loss in keratoconus and to apply appropriate interventions for timely preservation/enhancement of vulnerable visual functions.

## Introduction

Structure subserves function in biological systems; a modification in structure arising from normal development, aging, disease or iatrogeny may lead to commensurate changes in function.^1^ Studies on structure-function relationship are routinely carried out for ophthalmic diseases, with the most systematic ones assessing the relationship between the changes in optic nerve head/nerve fiber layer and visual field in glaucoma^2^^,^^3^ or the relationship between the structural integrity of the retina and visual functions like acuity, contrast sensitivity, and preferred retinal locus in age-related macular degeneration.^4^^,^^5^ Such systematic evaluation of structure-function relationship has seldom been performed for keratoconus; a progressive, bilateral disease that causes visual impairment due to distortions in the shape of the cornea.^6^^–^^8^ Changes in the optical structure of the cornea, the major refracting component of the eye, result in increased higher-order wavefront aberrations, and asymmetric corneal tomography/topography that significantly degrades the retinal image quality in keratoconus.^9^^,^^10^ These changes are quantified using several corneal tomographic indices, with the general understanding that multimetric indices that are based on a combination of change descriptors (e.g. D-index) are more reliable at identifying the disease and/or its progression than unimetric indices.^11^^,^^12^ Unlike structural loss, changes in visual function with disease progression are less understood. High contrast visual acuity is the most commonly used measure of visual function in keratoconus. Visual acuity progressively deteriorates with increasing disease severity,^8^^,^^13^ more so for uncorrected or spectacle-corrected conditions than with contact lenses.^14^^,^^15^ A few recent investigations on changes in contrast sensitivity^16^ and stereoacuity^17^^,^^18^ in keratoconus show similar trends to visual acuity.^15^ Although these studies provide useful insights into visual function loss in keratoconus, they do not describe the exact nature of the structure-function relationship in this disease. Therefore, the present study aimed to systematically determine changes in spatial vision (high-contrast visual acuity and contrast sensitivity) and depth vision (stereoacuity) with increasing severity of keratoconus and model the structure-function relationship to identify the phases of loss in visual function with disease severity. Knowledge of the structure-function relationship in keratoconus would enable superior care for the patient through (1) the prediction of vision loss in keratoconus at the point of care, (2) predict future rate of vision loss with disease progression, (3) assess the uniformity of loss across different domains of vision, and (4) apply targeted, evidence-based interventions for preserving or enhancing the vulnerable visual functions in a timely manner.

**Figure 1.**
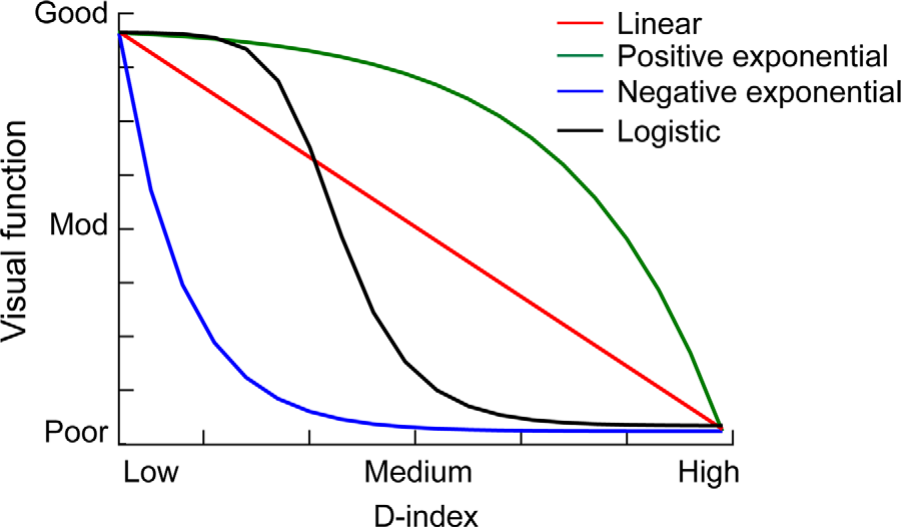
Schematic of the four patterns of structure-function relationship hypothesized in keratoconus. The four patterns were quantitatively described using regression equations described in the Methods section.

Structure-function relationship in human disease has been modeled in the past using linear and various nonlinear regression models.^19^^,^^20^ In ophthalmology, a mathematical formulation of this relationship is best described in glaucoma between the changes in retinal nerve fiber layer thickness or ganglion cell density and sensitivity losses during visual field examination.^21^ Inspired by these approaches, the present study hypothesizes that the structure-function relationship in keratoconus may follow one of four trends shown in Figure 1. First, visual function can deteriorate linearly with structural loss (see Fig. 1, red line). Second, the function may remain immune to structural loss at the beginning of the disease (the ceiling effect), followed by a monotonic deterioration with further losses in the structure (see Fig. 1, green curve). The ceiling effect may reflect the insensitivity of the visual function per se or its measurements to the underlying loss of structure. Third, the function may deteriorate monotonically from the beginning of the structural loss but may saturate beyond a certain disease severity (the floor effect; see Fig. 1, blue curve). The floor effect may represent the absolute minimum value that may be possible for that function (e.g. no form perception for visual acuity) or a certain level above the absolute minimum where the function asymptotes to. Fourth, the structure-function relationship may be a combination of the ceiling effect, followed by a monotonic deterioration and a floor effect thereafter (see Fig. 1, black curve). Based on our general understanding of contrast sensitivity being a more sensitive marker of vision loss in ophthalmic disease than high contrast acuity, including keratoconus,^22^^,^^23^ it was hypothesized that the structure-function relationship will show a more prominent ceiling effect and a slower loss rate thereafter for visual acuity compared to contrast sensitivity. Stereoacuity, on the other hand, is determined by both the overall and interocular difference in image quality of the two eyes.^24^^,^^25^ The latter is a stronger predictor of deteriorating stereoacuity than the former,^24^ especially when the stereo processing is driven by lower rather than higher spatial frequencies (the contrast or blur paradox).^26^ Based on this background, it was hypothesized that stereoacuity loss will follow one of the trends in Figure 1 for both an overall increase in disease severity and an increase in the interocular disease asymmetry. Keratoconic individuals with early and bilaterally similar disease will have better stereoacuity than those with advanced disease severity with or without bilateral symmetry.

## Methods

This cross-sectional and observational study was conducted at the L. V. Prasad Eye Institute (LVPEI), Hyderabad, India, between January 2021 and April 2022. The study adhered to the tenets of the Declaration of Helsinki, and it was approved by the Institutional Review Boards of LVPEI and City, University of London. All subjects signed a written informed consent form prior to study participation. All subjects were inducted into the study after being diagnosed with bilateral keratoconus for the first time by an experienced clinician following a thorough slit lamp examination, corneal tomography, and objective/subjective refraction. Subjects were included in the study if the clinical investigation revealed one or more of the following signs of keratoconus: scissoring reflex in retinoscopy; Munson's sign, cornea ectasia, Fleischer's ring, and Vogt's striae in slit lamp examination; steep curvature, asymmetry and/or skewing of the bow-tie pattern on curvature maps, increased elevation points in the Belin-Ambrosio enhanced ectasia display, and relative thinning of the cornea in Scheimpflug imaging tomography (Pentacam HR; Oculus Optikgeräte, Wetzlar, Germany).^27^ The severity of keratoconus was not graded at the time of study inclusion but was done so subsequently using the D-index, as described below. Subjects with scarred or inflamed corneas, other ocular pathology, history of contact lens wear, or ocular surgery were excluded. Comparison data from ten age-similar visually healthy controls were obtained from previous data collected in the laboratory for related experiments.

### Assessment of Corneal Structure

The D-index, assessed using Scheimpflug imaging tomography,^27^ was considered as the primary outcome measure of corneal structural deformation in this study. This metric has been shown to have good reliability in the diagnosis and progression of keratoconus.^28^ The D-index, derived for both eyes of all subjects using the Belin-Ambrósio enhanced ectasia display map, includes deviations of the front and back surface elevations of the cornea, pachymetric progression, thinnest corneal point, and deviation of Ambrósio relational thickness maximum.^11^ For keratoconus, higher D-index values indicated greater disease severity. Alternate indices of corneal tomographic deformation in keratoconus were also obtained in this study and a detailed comparison for the present cohort is described in Appendix I.^27^^,^^29^^,^^30^

### Assessment of Visual Function

#### High Contrast Visual Acuity and the Contrast Sensitivity Function 

All psychophysical measurements were conducted with the subject's natural pupils and with their best-corrected refraction at the spectacle plane. The sphero-cylindrical refractive error was finalized using the maximum plus for maximum visual acuity criterion of clinical subjective refraction for each subject. The monocular high contrast visual acuity of each eye was assessed using an electronic projection chart (Complog Clinical Vision Measurement Systems Ltd., UK).^31^ Each level of acuity was assessed using five letters, randomly selected from the complete range of Sloan optotypes. The letters were displayed on an LCD monitor (1680 × 1050 pixels; 80 cd/m^2^) at 3-meter viewing distance. The acuity was determined using a thresholding algorithm that terminated when three out of five letters were incorrectly identified.^32^ The acuity was quantified as the total number of optotypes correctly identified, with 0.02 logMAR units assigned per optotype.^32^

The monocular contrast sensitivity function (CSF) of each eye was measured using a modified version of the qCSF paradigm, implemented using the Psychtoolbox-3 interface in Matlab (Mathworks Inc., Natick, MA, USA).^33^^–^^35^ In this paradigm, subjects made 2 alternate forced choice judgments of the Gabor stimulus orientation presented at 45 degrees or 135 degrees on a luminance-calibrated CRT monitor (1280 × 1024 pixels, 85 cd/m^2^) from 1-meter viewing distance. The Gabor stimuli subtended 4 degrees × 4 degrees at the eye's nodal point at this viewing distance. The algorithm determined the CSF over a broad range of spatial frequency (1 to 50 cycles/degree [cpd]), within 100 trials, by varying the grating spatial frequency and contrast in an adaptive thresholding manner using a one-step-ahead search algorithm to evaluate the next trial's possible results. The bit-depth of the stimulus display on the CRT monitor was enhanced to facilitate fine contrast presentations using the Bits# stimulus processor (Cambridge Research Systems Ltd., Kent, UK) that was synchronized with the Psychophysics toolbox. The CSF was quantified using the cumulative area under the curve (AUCSF), the cutoff spatial frequency, and the sensitivity at 3 cpd spatial frequency.^15^ Higher values of AUCSF indicated a larger “visible area” for spatial vision, whereas changes in the cutoff spatial frequency and sensitivity at 3 cpd provided insights into the relative contributions of the high and low spatial frequency channels in defining this visible area.^36^

#### Stereoacuity

Stereoacuity was measured using random-dot stereograms implemented using the Psychtoolbox-3 interface in Matlab. The random-dot field subtended 7 degrees × 7 degrees at 0.5-meter viewing distance on the LCD monitor (1680 × 1050 pixels; 80 cd/m^2^). Subjects viewed these stereograms through a mirror stereoscope and made two-alternate forced choice judgments of the orientation of a rectangular bar appearing in crossed disparity within the random-dot field. The stimulus disparity was scaled to the subject's interpupillary distance and viewing distance, and the mirror angle in the stereoscope was adjusted to overcome any horizontal heterophoria.^32^ Based on prior knowledge of stereoacuity being poor in keratoconus,^17^^,^^32^ the initial disparity value of the adaptive staircase was set at 800 arc seconds for these subjects and at 100 arc seconds for controls. The disparity values were subsequently modulated in a 2-down-1-up fashion, with the step-size ranging from 50% to 0.5% of the initial disparity value and terminating after 16 reversals. The average of the last 13 reversals was considered as the subject's stereoacuity.

### Assessment of the Structure-Function Relationship

To enable comparison of the structure-function relationship across the different measures of visual functions, all raw data were normalized against the corresponding values of controls before curve fitting. Normalization was achieved by dividing the visual function value of cases by the median value of that visual function obtained from controls. A normalized value of unity indicated that the performance of cases equaled that of controls. For visual acuity and AUCSF, normalized values less than unity indicated a poorer visual function in cases, relative to controls. Worsening of stereoacuity was indicated by its normalized values being greater than unity. Although the normalization was straightforward for AUCSF and stereoacuity, it was rendered meaningless for zero or negative values of visual acuities on a logMAR scale (e.g. 0.0 logMAR would produce an indeterminate number during the normalization process). This was overcome by converting the logMAR values into decimal scale and then normalizing them against the median value of controls.

The four putative trends in the structure-function relationship for keratoconus were quantitatively assessed for each visual function tested. A linear trend is defined by:

(1) $$\begin{array}{c} y=y_{0}+ax \end{array}$$

where, y is the normalized visual function, *y_0_* the y-intercept, *a* the rate of loss of visual function, and x the D-index. Positive and negative exponential trends are defined by:

(2) $$\begin{array}{c} y=y_{0}-a\left[1+e^{\left(\frac{x}{b}\right)}\right] \end{array}$$

and

(3) $$\begin{array}{c} y=y_{0}+a\left[1+e^{\left(-\frac{x}{b}\right)}\right] \end{array}$$

where, the symbols have the same meaning as for Equation (1) but *a* is the maximum amplitude of change in visual function and *b* is the rate of loss of visual function. A logistic function was used to model the trend, where there is a ceiling and floor effect, using the following equation:

(4) $$\begin{array}{c} y=y_{0\;+\;\frac{a}{{\left[1+e^{b\left(x-x_{0}\right)}\right]}^{c}}} \end{array}$$

where y is the normalized visual function, *y_0_* the lower asymptote of the logistic fit, *a* is the height of the sigmoid between the asymptotes, *b* the rate of loss of visual function, x the D-index, *x_0_* the midpoint of the logistic fit, and *c* the sharpness of the edge roll-offs.

**Figure 2.**
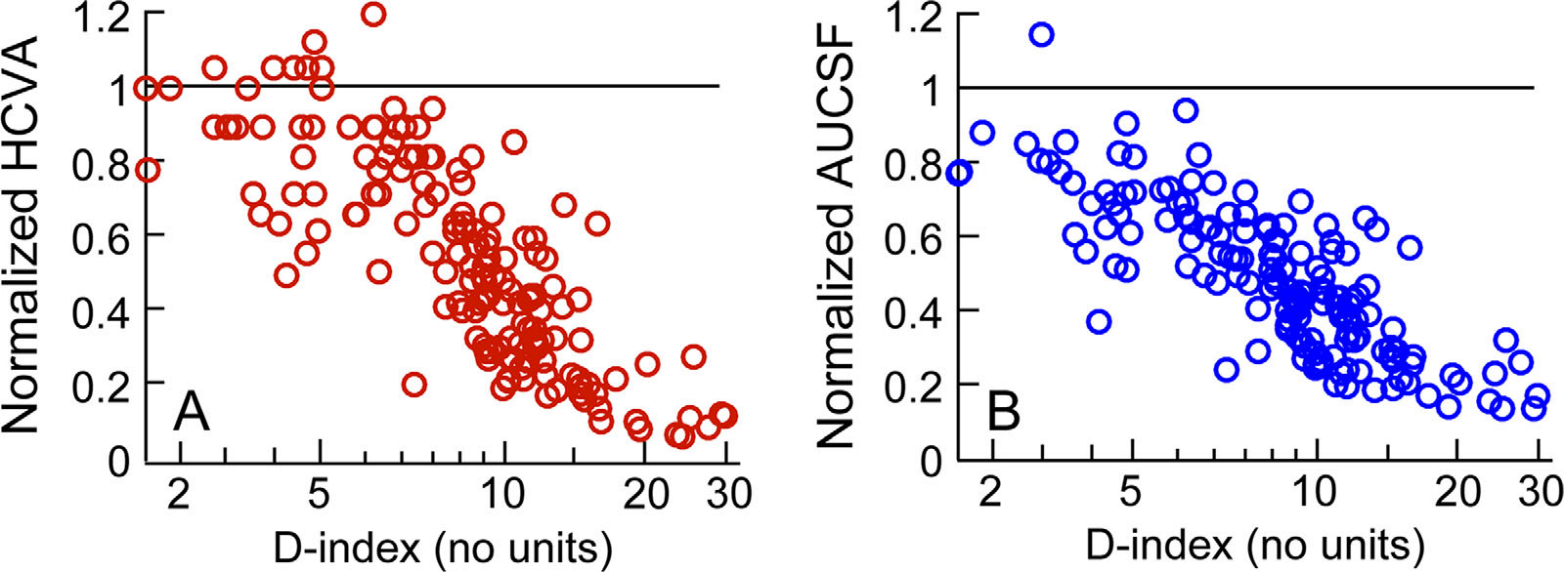
Scatter diagram of normalized spectacle-corrected high contrast visual acuity (HCVA; panel **A**) and normalized area under the CSF (AUCSF; panel **B**) plotted as a function of the D-index for all study subjects. The abscissa is plotted in logarithmic scale in both panels. Data points lying along the horizontal unity line indicate that the visual acuity and AUCSF of these subjects were equal to those of age-similar controls.

Curve fitting was performed in Matlab using the *fminsearch* algorithm that optimized the values of the free parameters using the Nelder-Mead multidimensional, unconstrained, nonlinear minimization process.^37^ The goodness of fit was assessed using the *R*^2^ values and the pattern of the residual errors obtained with the best-fit equation (Fig. 2). The first derivative of the best-fit equation described the rate of change of the given visual function per unit increase in D-index value.

### Statistical Analyses

All data were analyzed using Matlab R2017a and IBM SPSS statistics version 20.0 (SPSS, Chicago, IL, USA). Although monocular visual acuity and CSF data were obtained from both eyes of the subject, only data from the right eye was used for the analysis. The trend in the structure function relationship from the left eye were very similar to the right eye and hence is not reported here (Table 1). The Shapiro-Wilk test was used to determine the normality of the dataset and appropriate parametric or nonparametric tests were applied for analysis subsequently.

**Table 1.** Demographic, Refractive, and Topographic Details of Study Subjects

Age (y)	21 (15 to 38)	
Gender (M : F)	93 : 62	
	RE	LE
Steep keratometry (D)	51.10 (42 to 73.60)	51.40 (42 to 79.30)
Flat keratometry (D)	46.90 (41.60 to 66.40)	46.80 (41.40 to 71.30)
Maximum keratometry (D)	57.50 (42.60 to 88.40)	57.50 (42.50 to 91.20)
D-index (unitless)	9.02 (1.70 to 29.90)	8.80 (1.20 to 40.20)
M (D)	−3.00 (−0.50 to −33.00)	−2.90 (+1.60 to −28.00)
J0 (D)	0.00 (+5.20 to −6.00)	0.25 (+9.20 to −7.50)
J45 (D)	−0.91 (+2.50 to −5.90)	0.60 (+8.60 to −3.90)
HCVA (logMAR)	0.23 (1.10 to −0.12)	0.22 (1.60 to −0.15)
AUCSF (unit area)	1.10 (0.34 to 2.66)	1.10 (0.35 to 2.47)
Cutoff frequency (Cpd)	10.60 (2.90 to 33.70)	10.20 (2.40 to 27.70)
Sensitivity at 3 cpd (log sensitivity)	1.30 (0 to 2.30)	1.30 (0 to 2.30)
Stereoacuity (arc second)	487.40 (60.70 to 1667.90)	

The values indicate median (minimum to maximum) for each parameter described in the study. The M, J0, and J45 terms represent the sphero-cylindrical refractive error in power vectors, wherein M = spherical equivalent of refraction and J0 and J45 represent the regular and oblique astigmatic components of refraction.^38^

## Results

### Demographic, Refractive, and Topographic Features

A total of 155 subjects with keratoconus (310 eyes, age range = 15 to 38 years) were recruited within the study period (see Table 1). Visual acuity and CSF were successfully collected from all subjects whereas stereoacuity could be collected only from 140 subjects with keratoconus. Amongst the 15 subjects excluded, 3 of them were due to logistical barriers and the remaining 12 reported difficulty in understanding the task. Because the data were not normally distributed, all outcome parameters are reported as median values along with appropriate measures of data dispersion (see Table 1). Sphero-cylindrical refraction values are reported in standard power vector notation.^38^ Data from 10 age-similar controls were used for the normalization process. Median values (minimum to maximum range) of the control group were: −0.06 logMAR (range = −0.02 to −0.12 logMAR) for high contrast visual acuity; 2.35 units (range = 2.30 to 2.55 units) for AUCSF, 26 cpd (range = 25.2 to 35.2 cpd) for cutoff spatial frequency, 2.0 log sensitivity (1.95 to 2.30 log sensitivity) for sensitivity at the peak spatial frequency, and 32 arc seconds (range = 25.3 to 73.4 arc seconds) for stereoacuity.

### Overall Trends in the Structure-Function Relationship

#### Visual Acuity and Contrast Sensitivity

**Figure 3.**
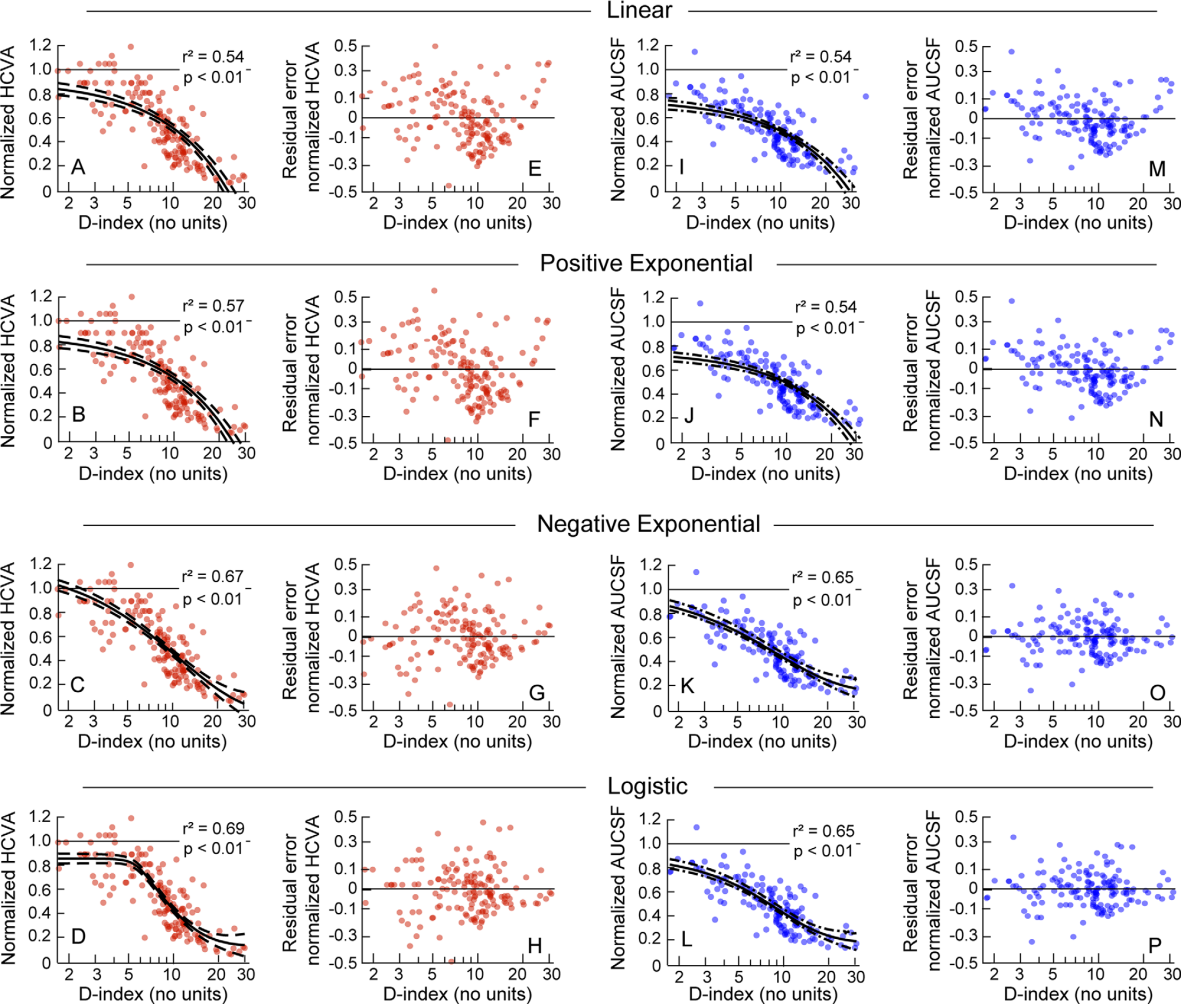
Same data as Figure 2 plotted along with the best-fit regression equations describing the pattern of structure-function relationship for high-contrast visual acuity (panels **A–D**) and area under CSF (AUCSF; panels **I–L**). The *dashed curves* around the best-fit curve indicate ±95% confidence interval of the fit. The residual errors of the fit are shown for visual acuity (panels **E–H**) and AUCSF (panels **M–P**). Other details are the same as Figure 2. Note that a logarithmic scale has been used for the x-axis, meaning a linear relationship appears nonlinear.

The normalized visual acuity and AUCSF deteriorated monotonically with increasing D-index before reaching their respective “floor” phases (see Fig. 2). A prominent “ceiling” phase was observed for visual acuity (see Fig. 2A) but not for AUCSF (all data points, except one, were well below the unity line even for early disease severities [see Fig. 2B]). The best-fit functions (Equations 1–4) for visual acuity and AUCSF are shown in Figure 3, with the adjusted *R*^2^ ranging from 0.54 to 0.69 and from 0.54 to 0.65 for acuity and AUCSF, respectively (both *P* < 0.01; see Fig. 3). Of the four fits, the linear regression equation had the lowest *R*^2^ for both visual acuity and AUCSF (see Figs. 3A, 3I) whereas the negative exponential equation and the logistic equation had the highest *R*^2^ values for both outcome variables (see Figs. 3C, 3D, 3K, 3L). The fit's residual errors for visual acuity and AUCSF showed a prominent underestimation bias for the mid-range of D-indices (D-index from 10–20) and a prominent overestimation bias for the high range of D-indices (D-index >20) for the linear regression equation and the positive exponential equation (see Figs. 3E, 3F, 3M, 3N). These biases were less prominent for the negative exponential equation for both outcome variables and least for the logistic equation (see Figs. 3G, 3O, 3H, 3P, Table 2). Both these functions also indicated differences in the rate of loss of visual acuity and AUCSF with increasing D-index; the former variable demonstrated a faster rate of decline than the latter (compare Figs. 3C and 3D with 3K and 3L). The logistic equation was meant to identify the “ceiling” and “floor” effects in the data (see Fig. 1, Equations 2, 3, and 4). However, the performance of the logistic equation was only marginally superior to the negative exponential equation for visual acuity but inferior for AUCSF reflecting the presence of a ceiling effect in the former but not in the latter (compare Figs. 3C and 3D with 3K and 3L, see Table 2). Both functions predicted the prominent floor effect equally well (see Fig. 3, Table 2).

**Table 2.** Coefficients of the Best-Fit Logistic Regression Equation for Spatial Vision Parameters Considered in this Study (Equation 4)

	x0	y0	y1	a	b	c
High contrast visual acuity	5.52	0.14	0.87	0.73	1.82	0.11
Area under CSF (area units)	0.92	0.19	1.22	1.03	0.27	0.57
Cut off spatial frequency (cpd)	−5.39	0.17	4.4	4.23	0.09	1.84
Sensitivity at 3 cpd	−13.14	−0.23	2.33	2.56	0.25	0.18

x0 = midpoint of the fit, y0 = lower asymptote of the fit, a = sigmoid height, b = rate of loss of visual function, and c = sharpness of the edge roll-offs. An additional parameter, y1, describes the value at the upper asymptote of the function. This parameter is derived by adding the lower asymptote to the height of the function.

#### Changes in the Cut-Off Spatial Frequency and Peak Sensitivity at 3 cpd with D-Index

**Figure 4.**
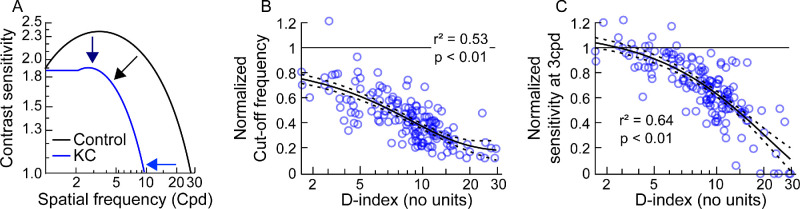
Panel (**A**) Representative contrast sensitivity functions (CSFs) from a control subject and a case with keratoconus. The attenuation of the CSF can occur from the loss of high spatial frequencies (*horizontal arrow*) or only from the loss of sensitivity at low spatial frequencies (*vertical arrow*) or from both (*diagonal arrow*). Panels (**B**, **C**) Scatter diagram of the normalized cut-off spatial frequency and sensitivity at 3 cpd plotted as a function of the D-index for the present cohort. The best-fit logistic regression equation along with the ±95% confidence interval is shown in these panels. All other details in these panels are the same as Figure 3.

The attenuation of AUCSF in keratoconus could occur due to a reduction in cut-off spatial frequency and/or a decline in sensitivity of lower spatial frequencies (Fig. 4A).^35^ The logistic regression equations showed a monotonic decline in performance for both these parameters, with similar loss rates with increased disease severity (see Figs. 4B, 4C, Table 2). The regression fit for normalized sensitivity at 3 cpd reached the level of controls for early disease severity, whereas this pattern was absent for the cutoff spatial frequency (see Figs. 4B, 4C, Table 2). The cutoff spatial frequency showed a floor effect at advanced disease severities, whereas such an effect was absent for the peak sensitivity at 3 cpd (see Figs. 4B, 4C). These results indicated that a reduction in the cutoff frequency precedes the loss of sensitivity in lower spatial frequencies. With advancing disease, the loss in cutoff spatial frequency asymptotes, while losses in the sensitivity at low spatial frequencies continues to decline. The loss of sensitivity in the lower spatial frequencies is also reflected in the peak spatial frequency of the CSF shifting from 6.2 to 1.6 cpd across the disease severities tested. However, no further analysis was undertaken on this parameter presently.

#### Stereoacuity

**Figure 5.**
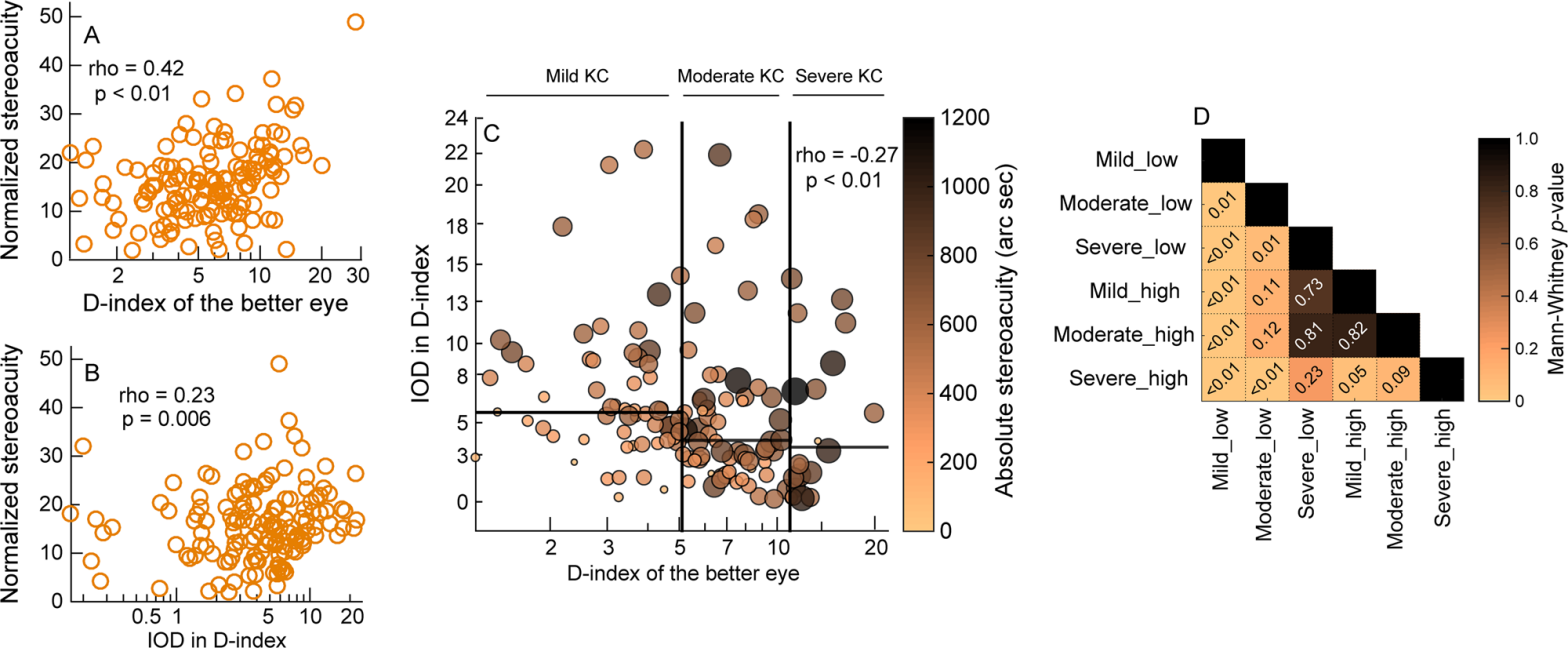
Panels (**A**, **B**) Scatter diagrams plotting the normalized stereoacuity as a function of the D-index of the better eye and interocular difference in D-index. Panel (**C**) Bubble plot showing the distribution of stereoacuity as a function of the D-index of the better eye and the interocular difference in D-index. The stereoacuity of each subject is indicated by the size and the color of the bubble; smaller sizes and lighter colors indicate better stereoacuity. Data points in this bubble plot are divided into separate groups of mild, moderate, and severe keratoconus in the better eye, using the Amsler-Krumeich classification scheme^39^ (*vertical lines* in panel **C**). The data points are also divided into lower-than-median (*_low) and higher-than-median (*_high) interocular difference in D-index for each severity group (horizontal lines in panel **C**). Panel (**D**) Heatmap of the statistical significance of the difference in stereoacuities between any two groups identified in panel **C** (Mann-Whitney *U* test).

Stereoacuity changes in keratoconus are determined by the overall structural loss in the cornea (represented here by the D-index of the better eye) and the difference in the structural loss between the two eyes (represented here by the interocular difference in D-index).^25^ Given the statistical independence of the two aforementioned measures of D-index for each pair of eyes in this cohort (Spearman's rho = −0.27, *P* < 0.01), the normalized stereoacuity was plotted independently as a function of the D-index of the better eye and the interocular difference in D-index in Figures 5A and 5B, respectively. The normalized values of stereoacuity fell well above the unity value in Figures 5A and 5B, reflecting the overall poorer stereoacuity in cases compared to controls (see Table 1). The normalized stereoacuities were significantly correlated with the D-index of the better eye (Spearman's rho = 0.42, *P* < 0.01) and the interocular difference in D-index (rho = 0.23, *P* = 0.006), even though both scatter diagrams showed significant intersubject variability in the data (see Figs. 5A, 5B). All four regression equations were poorly fit to the data (*R*^2^ ≤ 0.26, *P* ≥ 0.2), indicating that stereoacuity did not demonstrate a clear structure-function relationship in keratoconus explained by any of the four models used.

Qualitatively, the stereoacuity data of some cases were found to be significantly better than the median values noted above. To better understand this trend, a different approach was chosen to explore the relationship between the two independent variables that define bilateral image quality in the keratoconic eye (the D-index of the better eye and the interocular difference in D-index) and stereoacuity. The bubble plot shown in Figure 5C plots the stereoacuity of individual subjects as a function of both the D-index of the better eye and the interocular difference in D-index. In general, the smaller and lighter colored bubbles representing better stereoacuity were limited compared to the larger and darker colored bubbles, indicating an overall poor stereoacuity in the keratoconus cohort (Fig. 5C). To test the hypothesis set out earlier on the relation among D-index, interocular difference in D-index, and stereoacuity, the data points in the bubble plot were further divided into separate groups of mild, moderate, and severe keratoconus in the better eye, as described using the Amsler-Krumeich classification scheme for keratoconus severity (see Fig. 5C).^39^ The data points were further subdivided into those with lower-than- and higher-than-median interocular difference in D-index within each severity group (see Fig. 5C, Table 3). The smaller and lighter-colored bubbles were prominently placed in the group containing subjects with mild keratoconus in the better eye and with smaller interocular difference in disease severity, indicating better stereoacuity in this group, relative to the other groups (see Fig. 5C, Table 3). The stereoacuity of this group was significantly different from all other groups (see Fig. 5D). The stereoacuity of those with moderate severity of keratoconus in the better eye and low interocular difference in disease severity was significantly different from those with severe keratoconus and low or high interocular difference in disease severity (see Fig. 5D). All other comparisons were not statistically significant, indicating an upper limit saturation of stereoacuity in these groups (see Fig. 5D). Taken together, these findings indicate that stereoacuity is very vulnerable to the loss of optical structure in keratoconus and it tends to reach the floor level of performance much earlier than spatial vision.

**Table 3.** Median (25th to 75th Interquartile Range) of Stereoacuity (Arc Sec) for the Subjects With Mild, Moderate, or Severe Keratoconus in the Better Eye and With Lower-Than Median or Higher-Than Median Interocular Difference in disease severity

	D-index of the better eye		
	Mild	Moderate	Severe
IOD in D-index			
Lower-than median	247.6 (181.6 to 376.0)	427.1 (288.9 to 538.7)	547.6 (341.2 to 707.0)
Higher-than median	490.9 (406.8 to 600.5)	500.6 (381.4 to 638.2)	618.6 (487.7 to 758.9)

## Discussion

### Summary of Results

i) Spatial vision loss in keratoconus may be best described using a logistic nonlinear regression equation. In this model, high-contrast visual acuity shows a distinct ceiling effect at early stages of keratoconus, whereas contrast sensitivity deteriorates from the very beginning of the disease. The loss rate of visual acuity appears steeper than that of contrast sensitivity.
ii) The loss in CSF is due to a reduction in both the cutoff spatial frequency and the sensitivity of lower spatial frequencies, with the loss in the former parameter preceding the loss in the latter.
iii) Stereoacuity does not show a strong structure-function relationship in keratoconus. However, subjects with mild and bilaterally symmetric keratoconus show slightly better stereoacuity than those with moderate and advanced disease, with or without interocular symmetry.
iv) The structure-function relationship demonstrated here is not unique to the D-index. Several other corneal tomographic indices appear to capture veridically this structure-function relationship, albeit with some differences in the pattern of loss between indices.

### Implication for the Clinical Management of Keratoconus

The study findings clearly highlight the complex nature of the structure-function relationship in keratoconus. Several features of this relationship indicate the need to evaluate multiple visual functions and at multiple time points during disease progression to obtain a comprehensive understanding of vision loss in keratoconus (Figs. 2–5).^40^^–^^42^ Three features are of relevance here. First, the nonlinear nature of the structure-function relationship demonstrates that a given quantum of deterioration in optical structure does not result in an equal proportion of loss in function at all disease severities. Second, different visual functions may have different levels of vulnerability to the same loss of optical structure in keratoconus. Consequently, the loss observed in one visual function at a given stage of the disease cannot be extrapolated to other visual functions in a meaningful manner. Third, the differences in the pattern of the structure-function relationship observed across tomographic indices suggest that switching between indices to describe vision loss in keratoconus is not straightforward and not recommended for clinical practice (Fig. A1). With all these factors considered, the structure-function relationships derived in this study will help eye care practitioners make more meaningful inferences about the nature of vision loss experienced by patients with different disease severities, develop more targeted, evidence-based, interventions to preserve or enhance vulnerable visual functions, monitor more resilient functions for potential changes over time, and help patients better understand their condition, manage their expectations regarding activities of daily living, and enable them to make informed decisions about various treatment options and lifestyle changes, if required. For instance, the link between stereoacuity loss and interocular differences in keratoconus severity (see Fig. 5), suggests that clinical interventions in early to moderate keratoconus should incorporate approaches that will optimize binocularity by making the optical quality of both eyes similar, in addition to the conventional optimization of monocular visual acuities in each eye independently.^18^^,^^43^

The present study showed that AUCSF deteriorated in early keratoconus, even though high contrast visual acuity remained relatively intact (see Figs. 2^3^–4, Table 2). These results agree with Shneor et al.^22^ and Xian et al.^16^ who observed significant losses in the discrete spatial frequencies they probed within the CSF in patients with forme-fruste or subclinical keratoconus with intact visual acuities. This result can be interpreted in three ways. One, the loss of optical fidelity in early keratoconus is not large enough to deteriorate resolution of high spatial frequency optotypes used in the assessment of visual acuity, but they are large enough to impact contrast detection that attenuate the AUCSF. This explanation is counterintuitive because the cutoff spatial frequency in the CSF, an oft-used surrogate of the visual resolution limit,^35^ showed no such “ceiling” effect in our cohort (see Fig. 5B). Perhaps, the “ceiling effect” in high contrast visual acuity arises from the keratoconic eye's ability to correctly interpret a slightly blurred image, even while the associated retinal image quality loss produces deficiencies in contrast processing at threshold.^18^^,^^44^ Two, the measurement resolution of visual acuity is not as fine as that of contrast sensitivity, leading to a spurious “ceiling” effect for this visual function. That the ceiling effect was observed despite the standard protocol of recording visual acuities through a letter-by-letter allocation of acuity scores suggests that the ceiling effect is unlikely to reflect insensitivities in acuity recording. Three, even while all the experiments were conducted with the subject's best corrected spectacle refraction, it is possible that residual defocus and astigmatism remained uncorrected in their eyes owing to the overall variability in determining the end point of subjective refraction in keratoconus.^45^^,^^46^ This residual blur may have had a greater impact on contrast sensitivity than on visual acuity, thus leading to an earlier loss in the former parameter with disease severity than the latter (see Figs. 2–4, Table 2). This issue may be turned around to ask if the high variability of subjective refraction in keratoconus is a result of using high contrast visual acuity as the measure of its end point? Given the overall “crudeness” of high contrast visual acuity as a measure of spatial vision, perhaps using measures of contrast sensitivity or low contrast acuity to optimize subjective refraction may yield less variable results. To the best of our knowledge, this has not been empirically tested, but is worth investigating in the future. Whatever the reason for this effect, the results indicate that the eye care practitioner should not rule out disease progression or visual function loss if visual acuity remains unaltered – instead, visual acuity assessment should be complemented with assessments of CSF and stereoacuity in all patient visits. The study also observed that the acuity loss, once initiated beyond the ceiling phase, occurs at a rate that is steeper than AUCSF (see Fig. 4, Table 2). This results in both functions reaching the “flooring” phase at more or less the same magnitude of disease severity, even though the acuity loss started happening at a later level of disease severity (see Fig. 4). This result is somewhat unexpected because the distinct “ceiling” phase and an ability to recognize optotypes despite a loss of optical fidelity would have predicted the opposite trend. Alternately, this loss rate in acuity may indeed be what is predicted by optical quality loss, and the shallower loss rate of AUCSF reflects some form of active recalibration in contrast processing to optimize the “visible area” constituted by the CSF. Recently, reports on suprathreshold contrast processing certainly allude to the presence of such a recalibration in the keratoconic visual system.^47^^,^^48^

### Modeling the Structure-Function Relationship Data

Mathematical modeling of the raw data using regression analysis allows a determination of the extent to which the independent variable (D-index, in this case) explains the variability seen in the dependent variable (visual functions, in this case; see Figs. 3, 4).^2^^,^^30^ The residual plots indicates the presence of systematic biases in the way the regression fit explains the relationship between the dependent and independent variables (see Fig. 3).^37^ Distinct differences were observed among the four regression models tested in this study. The linear and positive exponential regressions could not veridically represent the transition of visual acuity and AUCSF into their respective flooring phases with increasing D-index values (see Fig. 3). The other two functions – the negative exponential and the logistic regression equations – explained more of the data variance, with little or no bias in the residual plots (see Fig. 3). Between these two functions, the logistic regression faired marginally better than the negative exponential function owing to its potential ability to delineate the three distinct phases in the structure-function relationship – the ceiling phase at early disease stages, the intermediate phase where functional loss is proportional to the structural loss, and the flooring phase at advanced disease stages. Both functions are equally well-suited to explain the latter two phases of the structure-function relationship, but the negative exponential is less suited to explain the ceiling effect relative to the logistic regression function (see Fig. 1). That the superiority of the logistic model was only marginal (albeit only for visual acuity; see Fig. 3), suggests that the early ceiling phase of this structure-function relationship is not as robust as the other two phases and may be subject to change with sample size and data variance. Two variations to the present regression models may be explored in the future. First, the data may be modeled using orthogonal regression analyses wherein the observed variance is evenly distributed between the dependent and independent variables to reflect the measurement variability inherent in both measures.^49^ That an ordinary regression analysis was performed here may be a limitation of this study, in this context. Second, the structure-function relationship in keratoconus may also be examined using piece-wise regression equations that will allow the visual function to be binned into distinct phases of disease severity.^50^ However, such an approach oversimplifies the structure-function relationship by artificially disrupting the continuous nature of the independent variables used in this analysis. Last, the asymmetric nature of disease presentation in keratoconus implies that the structure-function relationship described here may also be determined by choosing the eye with lesser disease severity. After all, naturalistic binocular viewing may be weighted in favor of the eye with lesser disease severity in asymmetric keratoconus.^18^ This approach was, however, not chosen for it may significantly restrict the range of disease severities over which this relationship is determined. Choosing only one eye, irrespective of its severity, addresses the correlation-bias that may be introduced in the data by choosing both eyes of the subject while retaining the desired range of disease severities needed to derive a meaningful structure-function relationship.^51^

### The Eye's Optical Quality as the Basis for the Loss in Visual Functions in Keratoconus

**Figure 6.**
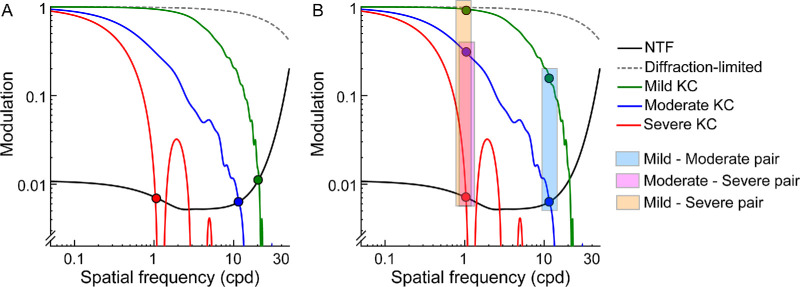
Rotationally averaged modulation transfer functions (MTFs) for diffraction-limited optics and for representative subjects with mild, moderate, and severe keratoconus (KC). Rotationally-averaged MTF's were derived from eye's higher-order aberrations (lower-order aberrations are assumed to be fully corrected) over 3 mm pupil diameter and for 555 nm light. Multiple lobes in the MTF of the severe KC represent phase shifts in the retinal image. The neural transfer function (NTF) was derived for a 25 year old subject at 400 Td of retinal illuminance, as described by Hastings et al.^57^ Panel (**A**) highlights the intersection point between the MTF and NTF for different severities of keratoconus (*filled circles*). Panel (**B**) depicts the matching spatial frequency and the associated interocular contrast differences for representative bilaterally asymmetric keratoconic cases (mild-moderate, mild-severe, and moderate-severe). The *filled circles* indicate the intersection between the MTF and NTF, representing the highest spatial frequency available for acuity and stereo processing.

The losses in spatial and depth vision observed in this study may be explained by the underlying loss of retinal image quality in the two eyes. Retinal image quality may be derived from the wavefront aberrations of the eye and quantified in terms of the modulation and phase transfer functions (MTF and PTF). The low-pass filtering of the MTF increases with disease severity and intersects the neural transfer function (NTF) at progressively lower spatial frequencies (Fig. 6). Consequently, the “visible” area for spatial vision and the contrast energy available at lower spatial frequencies get attenuated with increasing disease severity (see Fig. 6A).^52^ This may explain the progressive loss of monocular visual acuity and parameters of the CSF in the empirical data (see Figs. 2^3^–4). For the same reason, the highest spatial frequency available in the two eyes for correspondence matching and retinal disparity calculation progressively shifts to lower values with increasing disease severity (compare blue bar with red and orange bars in Fig. 6B). Stereo thresholds mediated through lower-spatial frequencies are poorer than those mediated through higher-spatial frequencies,^25^^,^^53^ predicting an overall worsening of stereoacuity with advancing keratoconus (see Fig. 6B). This loss is further compounded in those with bilaterally asymmetric disease by the underlying differences in contrast energy between the two eyes at the matching spatial frequency (the contrast/blur paradox effect in stereoacuity).^26^ Subjects with large interocular asymmetry in disease severity stand to lose more than those with milder and symmetric disease forms in the two eyes (compare blue bar with red and orange bars in Fig. 6B). The empirical data more or less reflected these predictions – stereoacuities progressively worsened with increasing disease severity for all sub-cohorts of keratoconus (see Table 3) and they were relatively better in sub-cohorts with lower-than-median interocular difference in D-index, than in those with higher-than-median interocular difference's (see Table 3).

### Future Considerations

The present study results may be extended in five possible directions. First, the logistic regression equation described here for the cross-sectional data may be extended to forecast longitudinal losses in spatial vision with natural progression of keratoconus. Obtaining such a dataset may not be trivial, for the disease progression is usually aggressively managed through surgical interventions such as collagen crosslinking to maintain visual quality in the patient.^54^ Second, the regression analysis described here was determined using structural indices that do not explicitly consider the morphology or the location of the cone in keratoconus.^55^ These parameters may influence the visual quality of the patient and, hence, they also need to be incorporated into models of structure-function relationship in this disease condition. Third, the structure-function relationship established in this study may be extended in the future through a detailed investigation of the retinal image quality loss induced by the corneal distortions in keratoconus.^10^^,^^56^ This endeavor may involve deriving appropriate image quality metrics from the wavefront aberration profile of subjects for viewing conditions that are equivalent to those experienced during the psychophysical measurements in this study (e.g. pupil size, polychromatic light spectrum, and properties of neural processing).^57^^–^^59^ Fourth, spatial vision needs to be evaluated binocularly to gain insights into the habitual viewing experience of patients with different severities of keratoconus. This issue is rather pertinent for bilaterally asymmetric keratoconus, wherein the visual system is known to optimize spatial vision by weighting the binocular input in favor of the eye with better retinal image quality.^10^^,^^32^^,^^43^ Fifth, whereas structure subserves function, visual functions, in turn, subserve functional vision that enable humans to effectively interact with their environment. The extent to which functional vision is impaired (i.e. create activity limitations) in different severities of keratoconus may be investigated in the future using appropriate measures of functional vision (e.g. depth vision tasks like placing pegs on a pegboard^60^ and/or using patient-reported outcome measures questionnaires (e.g. Keratoconus Outcomes Research Questionnaire^61^). This is especially important considering the recent observation that the vision-related quality of life is worse in keratoconus than in other forms of ocular pathology (e.g. degenerative/vascular retinal pathology) and that these measures of quality of life are poorly correlated with clinical measures of visual functions (e.g. high contrast visual acuity).^61^^,^^62^

## Conclusions

Vision loss in keratoconus is critically dependent on the overall disease severity in the two eyes, its bilateral symmetry (for binocular visual functions) and the type of visual function being evaluated. Among the visual functions commonly evaluated in the clinic, stereoacuity may be the first to deteriorate, followed by contrast sensitivity and then high contrast visual acuity. Losses in functional depth vision and contrast sensitivity may therefore be early markers of keratoconus, prior to perceptible losses in optical resolution. The logistic regression equation described in this study for cross-sectional data of spatial vision may be used for forecasting longitudinal losses vision loss with advancing keratoconus.

## Supplementary Material

Supplement 1

Supplement 2
