# Supplementary figures and images for: Structure-Function Relationship in Keratoconus: Spatial and Depth Vision
Source: Transl Vis Sci Technol. 2023 Dec 27;12(12):21. doi: 10.1167/tvst.12.12.21 (PMC10756247; doi:10.1167/tvst.12.12.21)

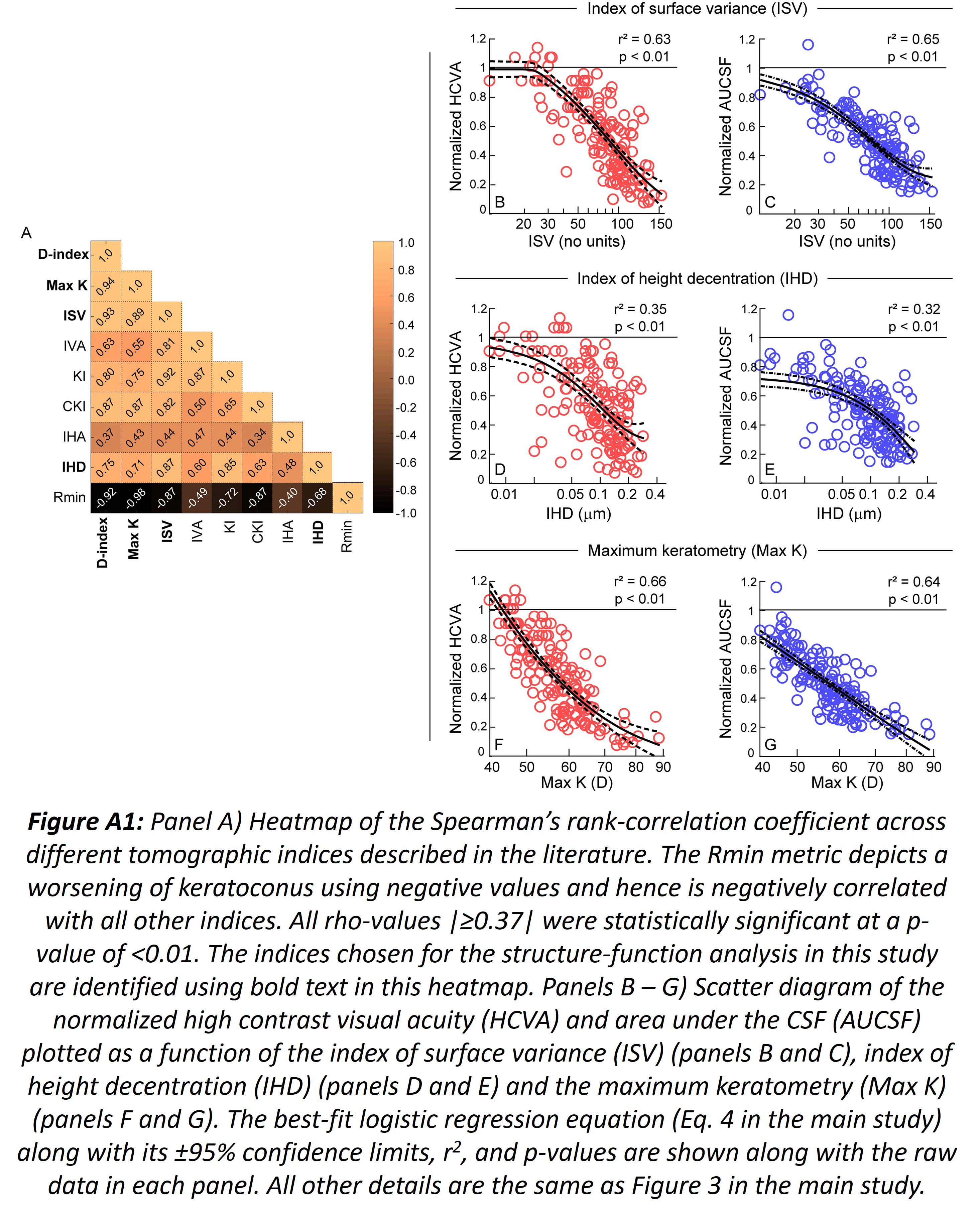

Supplement: Supplement 1 [file tvst-12-12-21_s001.jpg]
