# Supplementary material for: Structure-Function Relationship in Keratoconus: Spatial and Depth Vision
Source: Transl Vis Sci Technol. 2023 Dec 27;12(12):21. doi: 10.1167/tvst.12.12.21 (PMC10756247; doi:10.1167/tvst.12.12.21)
Supplement: Supplement 2 [file tvst-12-12-21_s002.docx]

**Appendix**

**Appendix 1: Structure-function relationship with other indices of change in corneal structure**

The D-index used in this study is one of the many tomographic indices of corneal structure for detection of keratoconus and its progression.^27^ Supplementary analyses were undertaken here to 1) determine the Spearman’s rank correlation coefficient between nine prominent indices obtained from the tomography map of all study subjects (Figure A1A) and 2) determine the pattern of the structure-function relationship for three specific indices from this list – the index of surface variance (ISV), the index of height decentration (IHD) and the maximum keratometry value (Max K) – that show high diagnostic accuracy in detecting keratoconus (ISV and IHD^30^) or is used commonly in clinics to ascertain the disease severity (Max K) (Figure A1B – G). The ISV is a unitless curvature-based index that represents the standard deviation of the sagittal radius of curvature of the cornea from its mean value.^30^ The IHD is a measure of the decentration of corneal elevation (in microns) in the vertical direction.^30^ The structure-function relationship was investigated only for high contrast visual acuity and AUCSF (and not for stereoacuity), based on the results found in the main study (Figures 3 and 5).

The tomographic indices showed a high Spearman’s rho value with D-index (rho≥ 0.75; p<0.001), except for two isolated instances involving the index of height asymmetry (rho: 0.37; p<0.001) and the index of vertical asymmetry (rho: 0.63; p<0.001) (Figure A1A). These results are in overall agreement with Kanellopoulos and Asimellis^30^ and reflect the comparable nature of sampling the corneal deformation with increasing keratoconus severity. Figure A1B – G plots the scatter diagram of the normalized high contrast visual acuity and AUCSF as a function of the ISV, IHD and Max K index values, along with the best-fit logistic regression equation. The r^2^ values of the fit for ISV and Max K were comparable to the D-index shown in the main study for both visual functions (Figure A1B, C, F and G). The r^2^ values for the IHD index were lower than that of the other three indices (Figures A1D and E). The ceiling effect in high contrast visual acuity was apparent for the ISV index, but not so for the IHD and Max K indices (Figures A1D and F). None of these indices showed a prominent floor effect for visual acuity (Figures A1B, D and F). The AUCSF trends showed no prominent ceiling or floor effect, and their y-intercept values were all lower than that of visual acuity for all three indices (Figures A1C, E and G). The loss rate in AUCSF was also lower than that of visual acuity (Figure A1B – G). These results indicate that the structure-function relationship described in the main study may be generalized across the different keratoconic indices described in the literature. Prominent variation in the structure-function trends do occur across indices, suggesting caution in using these indices interchangeably for the keratoconus detection/progression or for assessing structure-function relationships. These variations may reflect the measurement precision or the relative granularity with which these indices signify keratoconus severity (e.g., the Max K index may signify keratoconus severity with lesser granularity than the D-index). Such an analysis is, however, beyond the scope of the present study.
